# Supplementary material for: Adverse outcomes and mortality in users of non-steroidal anti-inflammatory drugs who tested positive for SARS-CoV-2: A Danish nationwide cohort study
Source: PLoS Med. 2020 Sep 8;17(9):e1003308. doi: 10.1371/journal.pmed.1003308 (PMC7478808; doi:10.1371/journal.pmed.1003308)
Supplement: S1 Appendix — (DOCX) [file pmed.1003308.s002.docx]

**S1 Appendix.** Detailed information on registries used in this study.

| registry | Citation | Information obtained | Variables |
| --- | --- | --- | --- |
| Danish National Prescription Registry (Læmiddelmiddelstatistik registeret) | Pottegård et al  (doi:10.1093/ije/dyw213) | All redeemed prescriptions from community pharmacies in the period 1995-2018 | ATC-code, fill date |
| Danish National Patient Registry (Landspatientregisteret) | Schmidt et al (doi:10.2147/CLEP.S91125) | In- and outpatient hospital diagnoses in the period 1977-2018 | ICD-10 code,  admission date, intensive care unit admission, mechanical ventilation, renal replacement therapy |
| Civil Registration System (CPR-registeret) | Schmidt et al  (doi:10.2147/CLEP.S179083) | Age, sex, migration- and vital status |  |
| The Danish Register of Causes of Death | Helweg-Larsen et al  (doi:10.1177/1403494811399958) | Date of death | Date of death |

ATC, anatomical therapeutical classification. ICD-10, international classification of diseases and related health problems 10^th^ revision
