# Supplementary material for: Adverse outcomes and mortality in users of non-steroidal anti-inflammatory drugs who tested positive for SARS-CoV-2: A Danish nationwide cohort study
Source: PLoS Med. 2020 Sep 8;17(9):e1003308. doi: 10.1371/journal.pmed.1003308 (PMC7478808; doi:10.1371/journal.pmed.1003308)
Supplement: S2 Appendix — (DOCX) [file pmed.1003308.s003.docx]

**S2 Appendix.** Codes used to define exposure, outcome, and covariate variables.

| **VARIABLE** | **CODING SYSTEM** | **CODE** |
| --- | --- | --- |
| *Exposures* |  |  |
| NSAID | ATC | M01A (excluding M01AX) |
| *Prescription drug use* |  |  |
| Antihypertensives | ATC | C08, C03CA, C07, C09 |
| Antidiabetic drugs | ATC | A10 |
| Low-dose aspirin | ATC | B01AC06, B01AC30, N02BA01 |
| Immunosuppressants | ATC | L04 |
| Opioids | ATC | N02A |
| Benzodiazepines | ATC | N05BA, N05CD |
| Z-drugs (Non-benzodiazepine benzodiazepine receptor agonists) | ATC | N05CF |
| 1st gen. antipsychotics | ATC | N05AA, N05AB, N05AC, N05AD, N05AF, N05AG, N05AL01 |
| 2nd gen. antipsychotics | ATC | N05AE, N05AH, N05AX, N05AL05 |
| Systemic glucocorticoids | ATC | H02AB |
| Inhaled corticosteroids | ATC | R03BA01-09, R03AK06-08, R03AK10, R03AK11 |
| *History of* |  |  |
| Asthma | ICD-10 | J45, J46 |
| Chronic obstructive pulmonary disease | ICD-10 | J41-J44 |
| Cardiovascular disease | ICD-10 | I20-I25, I47-I50 |
| Ischaemic stroke | ICD-10 | I63, I64 |
| Chronic kidney disease | ICD-10 | N00, N01, N03-N06, N08.8, N14.1, N14.2, N16.8, N17, N25.1, N26, N27 |
| Liver disease | ICD-10 | B18, K70.0- K70.3, K709, K71, K73, K74, K76.0, B15.0, B16.0, B16.2, B19.0, K70.4, K72, K76.6, I85 |
| Alcohol related disorders | ICD-10 | F10, E24.4, G31.2, G62.1, G72.1, I42.6, K29.2, K70, K85.2, K86.0, Q86.0, Z50.2, Z71.4, Z72.1 |
| Dementia | ICD-10 | F01-F04 |
| Cancer | ICD-10 | C00-C97, excluding C44 |
| Overweight or obesity | ICD-10 | E66 |
| Hemiplegia and paraplegia | ICD-10 | G04.1, G11.4, G80.1, G80.2, G83.0-G83.4, G83.9 |
| Osteoarthrosis | ICD-10 | M15-19 |
| Rheumatoid arthritis/collagen vascular disorders | ICD-10 | L94.0, L94.1, L94.3, M05, M06, M08, M12.0, M12.3, M30, M31.0 - M31.3, M32-M35, M45, M46.1, M46.8, M46.9 |
| Dysmenorrhoea | ICD-10 | N94.4-94.6 |
| *Other* |  |  |
| Phase of the outbreak | Calendar time | An indicator variable for whether the patient was tested before or after March 12, 2020 where the strategy changed from focusing on containment with contact tracing and testing to a mitigation strategy prioritizing testing of patients with severe symptoms, admitted patients, and health care professionals |
| *Outcomes* |  |  |
| Admission to intensive care unit | SKS | NABB, NABE |
| Mechanical ventilation | SKS | BGDA |
| Acute renal replacement therapy | SKS | BJFD0 |

ATC, anatomical therapeutical classification. ICD-10, international classification of diseases and related health problems 10^th^ revision. SKS, sundhedsvæsenets klassifikations system. NSAID, non-steroidal anti-inflammatory drugs. COPD, chronic obstructive pulmonary disease.
