# Supplementary material for: Adverse outcomes and mortality in users of non-steroidal anti-inflammatory drugs who tested positive for SARS-CoV-2: A Danish nationwide cohort study
Source: PLoS Med. 2020 Sep 8;17(9):e1003308. doi: 10.1371/journal.pmed.1003308 (PMC7478808; doi:10.1371/journal.pmed.1003308)
Supplement: S1 Table — (DOCX) [file pmed.1003308.s004.docx]

**S3 Table.** Univariate association with death (RRcd) and use of NSAIDs (RRce) for each covariate in the PS.

| **Covariate** | **RR(ce) (95% CI)** | ***p*-Value** | **RR(cd) (95% CI)** | ***p*-Value** |
| --- | --- | --- | --- | --- |
| Prescription drug use |  |  |  |  |
| Antihypertensives | 0.93 (0.67-1.30) | 0.67 | 1.44 (1.14-1.80) | < 0.01 |
| Antidiabetic drugs | 1.22 (0.79-1.86) | 0.37 | 1.60 (1.24-2.08) | < 0.001 |
| Low-dose aspirin | 0.85 (0.50-1.45) | 0.56 | 1.22 (0.94-1.59) | 0.13 |
| Immunosuppressants | 1.54 (0.48-4.94) | 0.47 | 1.49 (0.63-3.51) | 0.36 |
| Opioids | 2.38 (1.72-3.30) | < 0.001 | 2.18 (1.75-2.73) | < 0.001 |
| Benzodiazepines | 0.86 (0.42-1.77) | 0.68 | 2.70 (1.90-3.84) | < 0.001 |
| Z-drugs | 0.78 (0.41-1.50) | 0.46 | 1.60 (1.16-2.20) | < 0.01 |
| 1st gen. antipsychotics | 0.52 (0.07-3.78) | 0.52 | 3.53 (1.74-7.13) | < 0.01 |
| 2nd generation antipsychotics | 0.40 (0.13-1.27) | 0.12 | 3.52 (2.38-5.21) | < 0.001 |
| Systemic glucocorticoids | 1.40 (0.86-2.28) | 0.18 | 1.73 (1.28-2.33) | < 0.001 |
| Inhaled corticosteroids | 1.52 (1.01-2.29) | 0.05 | 1.41 (1.02-1.96) | 0.04 |
| Prior diagnoses |  |  |  |  |
| Asthma | 0.94 (0.56-1.57) | 0.81 | 1.15 (0.76-1.73) | 0.51 |
| COPD | 0.83 (0.44-1.56) | 0.56 | 1.80 (1.36-2.38) | < 0.001 |
| Cardiovascular disease | 0.57 (0.37-0.87) | < 0.01 | 1.42 (1.15-1.75) | < 0.01 |
| Stroke | 0.63 (0.31-1.25) | 0.19 | 1.15 (0.86-1.52) | 0.34 |
| Chronic kidney disease | 0.23 (0.03-1.65) | 0.14 | 2.67 (1.68-4.24) | < 0.001 |
| Liver disease | 1.05 (0.39-2.88) | 0.92 | 2.49 (1.33-4.66) | < 0.01 |
| Alcohol related disorders | 0.70 (0.29-1.72) | 0.44 | 2.20 (1.43-3.39) | < 0.001 |
| Dementia | 0.33 (0.08-1.34) | 0.12 | 1.66 (1.15-2.42) | < 0.01 |
| Cancer | 0.96 (0.60-1.53) | 0.85 | 1.37 (1.06-1.75) | 0.01 |
| Overweight or obesity | 1.55 (1.06-2.27) | 0.02 | 1.52 (1.08-2.16) | 0.02 |
| Hemiplegia and paraplegia | 0.91 (0.12-6.71) | 0.93 | 6.99 (2.65-18.42) | < 0.001 |
| Osteoarthrosis | 1.49 (1.05-2.11) | 0.03 | 1.01 (0.80-1.27) | 0.96 |
| Rheumatoid athritis | 1.82 (1.09-3.04) | 0.02 | 1.11 (0.76-1.64) | 0.58 |
| Dysmenorrhoea | 4.32 (1.94-9.63) | < 0.001 | - | - |

RRce, independent association between confounder and exposure (relative risk). RRcd, independent association between confounder and disease (relative risk). COPD, chronic obstructive pulmonary disease.
