# Supplementary material for: Adverse outcomes and mortality in users of non-steroidal anti-inflammatory drugs who tested positive for SARS-CoV-2: A Danish nationwide cohort study
Source: PLoS Med. 2020 Sep 8;17(9):e1003308. doi: 10.1371/journal.pmed.1003308 (PMC7478808; doi:10.1371/journal.pmed.1003308)
Supplement: S2 Table — (DOCX) [file pmed.1003308.s005.docx]

**S2 Table.** Association between current NSAID use and mortality, hospitalization, ICU admission, mechanical ventilation, and renal replacement therapy using an outcome assessment window of 60 days for mortality and 30 days for secondary outcomes.

| **Outcome** | **NSAID Users*** | | **Non-users** | | **Comparison** |  |  |  |
| --- | --- | --- | --- | --- | --- | --- | --- | --- |
|  | **Number of events/sample size** | **Risk (%)  (95% CI)** | **Number of events/sample size** | **Risk (%) (95% CI)** | **Risk difference (%) (95% CI)** | ***p*-Value** | **Risk ratio  (95% CI)** | ***p*-Value** |
| **Unmatched cohort** | | | | | | | | |
| Death | 14/248 | 5.6 (2.8-8.5) | 564/8988 | 6.3 (5.8-6.8) | -0.6 (-3.5-2.3) | 0.67 | 0.90 (0.54-1.51) | 0.69 |
| Hospitalization** | 57/228 | 25.0 (19.4-30.6) | 1512/8414 | 18.0 (17.1-18.8) | 7.0 (1.3-12.7) | 0.02 | 1.39 (1.11-1.75) | < 0.01 |
| ICU admission** | 12/247 | 4.9 (2.2-7.5) | 291/8956 | 3.2 (2.9-3.6) | 1.6 (-1.1-4.3) | 0.24 | 1.50 (0.85-2.63) | 0.16 |
| Mechanical ventilation** | 10/248 | 4.0 (1.6-6.5) | 232/8970 | 2.6 (2.3-2.9) | 1.4 (-1.0-3.9) | 0.25 | 1.56 (0.84-2.90) | 0.16 |
| Renal replacement therapy** | n<5/248 | -*** | -*** | -*** | 0.4 (-1.0-1.8) | 0.57 | 1.49 (0.47-4.69) | 0.50 |
| **Matched cohort** |  |  |  |  |  |  |  |  |
| Death | 14/224 | 6.3 (3.1-9.4) | 62/896 | 6.9 (5.1-8.7) | -0.7 (-4.3-3.0) | 0.72 | 0.90 (0.51-1.60) | 0.73 |
| Hospitalization | 50/204 | 24.5 (18.6-30.4) | 183/826 | 22.2 (19.0-25.3) | 2.4 (-4.3-9.1) | 0.49 | 1.11 (0.84-1.46) | 0.48 |
| ICU admission | 12/223 | 5.4 (2.4-8.3) | 42/889 | 4.7 (3.2-6.2) | 0.7 (-2.7-4.0) | 0.70 | 1.14 (0.60-2.16) | 0.69 |
| Mechanical ventilation | 10/224 | 4.5 (1.8-7.2) | 35/891 | 3.9 (2.5-5.3) | 0.5 (-2.5-3.6) | 0.73 | 1.14 (0.56-2.30) | 0.72 |
| Renal replacement therapy | n<5/224 | -*** | -*** | -*** | -0.3 (-2.1-1.5) | 0.71 | 0.80 (0.22-2.85) | 0.73 |

NSAID, non-steroidal anti-inflammatory drugs. ICU, intensive care unit.

*NSAID use was defined as a filled prescription within 30 days prior to the date cohort entry.

** Patients with a secondary outcome occurring during the exclusion assessment window were excluded, resulting in exclusion of *n* = 594 patients for hospitalisation, *n* = 33 for ICU-admission, *n* = 18 for mechanical ventilation, and  *n* = 6 for renal replacement therapy in unmatched cohorts and *n* = 90, 8, 5 and *n* < 5 in matched cohorts.

*** Censored to preserve anonymity for counts *n* < 5
