# Supplementary material for: Adverse outcomes and mortality in users of non-steroidal anti-inflammatory drugs who tested positive for SARS-CoV-2: A Danish nationwide cohort study
Source: PLoS Med. 2020 Sep 8;17(9):e1003308. doi: 10.1371/journal.pmed.1003308 (PMC7478808; doi:10.1371/journal.pmed.1003308)
Supplement: S3 Table — (DOCX) [file pmed.1003308.s006.docx]

**S3 Table.** Association between use of NSAIDs within 60 days before cohort entry and 30-day mortality, hospitalization, ICU admission, mechanical ventilation, and renal replacement therapy.

| **Outcome** | **NSAID users*** | | **Non-users** | | **Comparison** |  |  |  |
| --- | --- | --- | --- | --- | --- | --- | --- | --- |
|  | **Number of events/sample size** | **Risk (%) (95% CI)** | **Number of events/sample size** | **Risk (%) (95% CI)** | **Risk difference (%)  (95% CI)** | ***p*-Value** | **Risk ratio (95% CI)** | ***p*-Value** |
| **Unmatched cohort** | | | | | | | | |
| Death | 24/445 | 5.4 (3.3-7.5) | 511/8791 | 5.8 (5.3-6.3) | -0.4 (-2.6-1.7) | 0.70 | 0.93 (0.62-1.38) | 0.71 |
| Hospitalization** | 90/406 | 22.2 (18.1-26.2) | 1422/8236 | 17.3 (16.4-18.1) | 4.9 (0.8-9.0) | 0.02 | 1.28 (1.06-1.55) | < 0.01 |
| ICU admission** | 20/443 | 4.5 (2.6-6.5) | 270/8760 | 3.1 (2.7-3.4) | 1.4 (-0.5-3.4) | 0.15 | 1.46 (0.94-2.28) | 0.09 |
| Mechanical ventilation** | 17/444 | 3.8 (2.0-5.6) | 218/8774 | 2.5 (2.2-2.8) | 1.3 (-0.5-3.2) | 0.15 | 1.54 (0.95-2.50) | 0.08 |
| Renal replacement therapy** | n<5/445 | -*** | -*** | -*** | 0.3 (-0.6-1.1) | 0.58 | 1.39 (0.50-3.80) | 0.53 |
| **Matched cohort** | | | | | | | | |
| Death | 21/402 | 5.2 (3.0-7.4) | 83/1608 | 5.2 (4.0-6.3) | 0.1 (-2.4-2.5) | 0.96 | 1.01 (0.63-1.63) | 0.96 |
| Hospitalization | 79/370 | 21.4 (17.2-25.5) | 261/1493 | 17.5 (15.2-19.7) | 3.9 (-0.9-8.6) | 0.11 | 1.22 (0.97-1.54) | 0.09 |
| ICU admission | 19/400 | 4.7 (2.7-6.8) | 62/1598 | 3.9 (2.8-4.9) | 0.9 (-1.5-3.2) | 0.47 | 1.22 (0.73-2.05) | 0.44 |
| Mechanical ventilation | 16/401 | 4.0 (2.1-5.9) | 52/1603 | 3.2 (2.3-4.2) | 0.7 (-1.4-2.9) | 0.50 | 1.23 (0.70-2.16) | 0.47 |
| Renal replacement therapy | n<5/402 | -*** | -*** | -*** | 0.1 (-1.1-1.2) | 0.91 | 1.07 (0.34-3.34) | 0.91 |

NSAID, non-steroidal anti-inflammatory drugs. ICU, intensive care unit.
*NSAID use was defined as a filled prescription within 60 days prior to the date cohort entry.

** Patients with a secondary outcome occurring during the exclusion assessment window were excluded, resulting in exclusion of *n* = 594 patients for hospitalisation, *n* = 33 for ICU-admission, *n* = 18 for mechanical ventilation, and *n* = 6 for renal replacement therapy in unmatched cohorts and *n* = 147, 12, 6 and *n* < 5 in matched cohorts.

*** Censored to preserve anonymity for counts *n* < 5
