# Supplementary material for: Adverse outcomes and mortality in users of non-steroidal anti-inflammatory drugs who tested positive for SARS-CoV-2: A Danish nationwide cohort study
Source: PLoS Med. 2020 Sep 8;17(9):e1003308. doi: 10.1371/journal.pmed.1003308 (PMC7478808; doi:10.1371/journal.pmed.1003308)
Supplement: S4 Table — (DOCX) [file pmed.1003308.s007.docx]

**S4 Table.** Association between NSAID prescription fills within the period 60 days to 14 days before cohort entry and 30-day mortality, hospitalization, ICU admission, mechanical ventilation, and renal replacement therapy

| **Outcome** | **NSAID users*** | | **Non-users** | | **Comparison** |  |  |  |
| --- | --- | --- | --- | --- | --- | --- | --- | --- |
|  | **Number of events/sample size** | **Risk (%) (95% CI)** | **Number of events/sample size** | **Risk (%)** | **Risk difference (95% CI)** | ***p-*Value** | **Risk ratio  (95% CI)** | ***p*-Value** |
| **Unmatched cohort** | | | | | | | | |
| Death | 17/350 | 4.9 (2.6-7.1) | 518/8886 | 5.8 (5.3-6.3) | -1.0 (-3.3-1.3) | 0.41 | 0.83 (0.52-1.33) | 0.45 |
| Hospitalization** | 67/319 | 21.0 (16.5-25.5) | 1445/8323 | 17.4 (16.5-18.2) | 3.6 (-0.9-8.2) | 0.12 | 1.21 (0.97-1.50) | 0.09 |
| ICU admission** | 16/348 | 4.6 (2.4-6.8) | 274/8855 | 3.1 (2.7-3.5) | 1.5 (-0.7-3.7) | 0.19 | 1.49 (0.91-2.43) | 0.12 |
| Mechanical ventilation** | 13/349 | 3.7 (1.7-5.7) | 222/8869 | 2.5 (2.2-2.8) | 1.2 (-0.8-3.2) | 0.23 | 1.49 (0.86-2.58) | 0.16 |
| Renal replacement therapy** | n<5/350 | -*** | -*** | -*** | 0.2 (-0.8-1.2) | 0.68 | 1.31 (0.41-4.17) | 0.64 |
| **Matched cohort** | | | | | | | | |
| Death | 14/312 | 4.5 (2.2-6.8) | 74/1248 | 5.9 (4.6-7.3) | -1.4 (-4.1-1.2) | 0.29 | 0.76 (0.43-1.33) | 0.33 |
| Hospitalization | 60/287 | 20.9 (16.2-25.6) | 191/1158 | 16.5 (14.2-18.8) | 4.4 (-0.8-9.7) | 0.10 | 1.27 (0.97-1.65) | 0.08 |
| ICU admission | 15/310 | 4.8 (2.4-7.2) | 50/1241 | 4.0 (2.8-5.2) | 0.8 (-1.9-3.5) | 0.55 | 1.20 (0.68-2.13) | 0.53 |
| Mechanical ventilation | 12/311 | 3.9 (1.7-6.0) | 42/1244 | 3.4 (2.3-4.5) | 0.5 (-1.9-2.9) | 0.69 | 1.14 (0.60-2.17) | 0.68 |
| Renal replacement therapy | n<5/312 | -*** | -*** | -*** | -0.1 (-1.3-1.2) | 0.90 | 0.92 (0.26-3.33) | 0.90 |

NSAID, non-steroidal anti-inflammatory drugs. ICU, intensive care unit.
*NSAID use was defined as a filled prescription within 60 days to 14 days prior to the date cohort entry.

** Patients with a secondary outcome occurring during the exclusion assessment window were excluded, resulting in exclusion of *n* = 594 patients for hospitalisation, *n* = 33 for ICU-admission, *n* = 18 for mechanical ventilation, and n=6 for renal replacement therapy in unmatched cohorts and  *n* = 115, 9, 5 and *n* < 5 in matched cohorts.

*** Censored to preserve anonymity for counts *n* < 5
