# Supplementary material for: Adverse outcomes and mortality in users of non-steroidal anti-inflammatory drugs who tested positive for SARS-CoV-2: A Danish nationwide cohort study
Source: PLoS Med. 2020 Sep 8;17(9):e1003308. doi: 10.1371/journal.pmed.1003308 (PMC7478808; doi:10.1371/journal.pmed.1003308)
Supplement: Attachment — Submitted filename: response-reviewer-comments-v4.docx [file pmed.1003308.s008.docx]

**Att. Emma Veitch, PhD
PLOS Medicine**

**RE: PMEDICINE-D-20-02753R1**

Dear Dr Veitch

Thank you for your letter concerning our manuscript. We appreciate the constructive and thoughtful comments provided by you and the reviewers and are grateful for the opportunity to send you a revised manuscript. We hope that we have addressed your concerns satisfactorily but remain open to further revision.
Please see below for our responses to editorial and reviewer comments and subsequent changes to the manuscript:

Requests from the editors:

* In the last sentence of the Abstract Methods and Findings section, please include a brief note regarding any key limitation(s) of the study methods overall.

**Author’s response and changes to the manuscript:**
We have added the following sentence regarding the main limitations of the study in the “Methods and findings” section of the abstract (page 3, line 54-59):
*“The main limitations of the study are possible exposure misclassification, as not all individuals who redeem an NSAID prescription will be using the drug continuously, and possible residual confounding by indication, as NSAIDs may generally be prescribed to healthier individuals due to their side effects, but on the other hand may be prescribed for early symptoms of severe COVID-19.”*

*At this stage, we ask that you include a short, non-technical Author Summary of your research to make findings accessible to a wide audience that includes both scientists and non-scientists. The Author Summary should immediately follow the Abstract in your revised manuscript. This text is subject to editorial change and should be distinct from the scientific abstract. Please see our author guidelines for more information: https://journals.plos.org/plosmedicine/s/revising-your-manuscript#loc-author-summary

# **Author’s response and changes to the manuscript:** We have added the following “Author summary” to the manuscript, see page 4: *“Why was this study done?*

- *During the early phases of the pandemic of coronavirus disease (COVID-19), concerns have been raised that ibuprofen, a drug commonly used to treat weak pain and fevers, may lead to a more severe course of coronavirus disease.*
- *If this risk is verified, it would be important to reduce the use of ibuprofen and ibuprofen-like drugs, commonly referred to as NSAIDs, among patients at risk of COVID-19.*

*What did the researchers do and find?*

- *We identified all Danish residents who were tested positive for the infectious agent of COVID-19 and grouped them into users and non-users of NSAIDs.*
- *The risks of being hospitalised, admitted to the intensive care unit or dying were compared between the two groups.*
- *Overall, risks for all studied outcomes were similar between users and non-users of ibuprofen and other NSAIDs.*

*What do these findings mean?*

- *NSAIDs do not lead to more severe coronavirus disease according to this study.*

*As noted by one reviewer, it would be good to include in the Discussion a more explicit and thorough discussion of the main limitations of the study methods to answer the question of interest.

**Author’s response and changes to the manuscript:**
We have added a clear “Strengths and limitations”(page 20, line 315) section to the discussion and added a short paragraph (page 20, line 323-327), listing the main limitations of the study, before explaining these in more detail:
*“The main limitations of our study are potential misclassification of non-users as NSAID users, potentially unmeasured confounding due to a lack of information on study participants’ bodyweight, an important risk factor of severe COVID-19, and confounding by indication due to NSAIDs possibly being prescribed due to early symptoms of severe COVID-19.”*

We have also revised the following section (p. 21-22, line 331-340) that originally read:
*“However, exposure misclassification may still be present since information on adherence, intended duration and dose was not available. Thus, the fact that a patient had filled a prescription for NSAIDs, may be considered an indicator of availability of NSAIDs rather than of actual use of NSAIDs.”*
which now reads
*“However, exposure misclassification may still be present since information on adherence, intended duration and dose was not available. Some patients will not be using NSAIDs continuously after filling a prescription, and consequently some of these patients will be incorrectly classified NSAID users at the time of a positive test for SARS-CoV-2. The fact that a patient had filled a prescription for NSAIDs, may be considered an indicator of availability of NSAIDs rather than of actual use of NSAIDs. As information on exposure status was collected prior to our outcomes of interest, the misclassification is nondifferential and may introduce a bias towards the null. This could lead to wrongly dismissing a causal detrimental effect of NSAIDs on the prognosis of COVID-19 if many NSAID users truly were non-users.”*

*As the paper reports analyses of data from a cohort study, it would be good to ensure that the study is reported according to the STROBE guideline; if doing this please include the completed STROBE checklist as Supporting Information. Please add the following statement, or similar, to the Methods: "This study is reported as per the Strengthening the Reporting of Observational Studies in Epidemiology (STROBE) guideline (SChecklist)." The STROBE guideline can be found here: http://www.equator-network.org/reporting-guidelines/strobe/. When completing the checklist, please use section and paragraph numbers, rather than page numbers.

**Author’s response and changes to the manuscript:**
We have added the following sentence to the beginning of the methods section (page 6, line 112-114):
*“This study is reported as per the Strengthening the Reporting of Observational Studies in Epidemiology (STROBE) guideline."*

-----------------------------------------------------------

Comments from the reviewers:

Reviewer #1: I greatly enjoyed reading this manuscript, which is very neatly written with the entire emphasis on solid statistical evaluation, as I would expect. Some of the English is cumbersome and sloppy (eg. abstract: "matched to up to...") but on the whole it is an excellent paper, and a useful addition to the literature.

Considering the methods used and the 'solidity' of the database I have some suggestions, and I am deliberately keeping this brief:

1. It would be helpful if statins could be studied too, using almost identical methods. The title would need to be changed to reflect this.

**Author’s response:**

We thank the reviewer for the suggestion. However, studying a potential association between statins and adverse outcomes in individuals infected with SARS-CoV-2 is beyond the scope of this paper.

2. In view of the RECOVERY data on dexamethasone, can steroids be looked at too? These data should at least be mentioned. As per 1.

**Author’s response:**

Similarly as for the suggestion above, this is considered beyond the scope of the present paper. Furthermore, there are ongoing efforts being made in Denmark to investigate both use of oral steroids as well as other immunomodulating agents in the same data sources used in this paper.

3. It was disappointing not to see a paragraph or 2, in the introduction and discussion, on mechanisms here that may be relevant and operational, leading the authors to study this as more than a simple correlation ie. is there any suggestion that the potential association may have been causative and problematic, based on the first line of the abstract? This is not expanded upon anywhere in the text and I do not see clinicians concerned any more about NSAID use (this is an old story as the authors know).

**Author’s response and changes to the manuscript:**
We have added a paragraph on the hypothesized mechanism by which NSAIDs could cause severe COVID-19 in relation to the findings in our study (page 23-24, line 367-379):
*“The lack of association, between use of NSAIDs and adverse outcomes in individuals tested positive for SARS-CoV-2, in our study could be explained by multiple factors. One, NSAIDs may not increase angiotensin converting enzyme 2 in humans. The original hypothesis stems from experiments conducted in diabetic rats [30] and the findings may not transfer between organisms. Two, an increased angiotensin converting enzyme 2 expression may not affect the risk of severe COVID-19. Studies on angiotensin converting enzyme inhibitors and angiotensin receptor blockers and the risk of contracting COVID19 or experiencing a severe course of disease have not shown any association [31-33]. Three, the adverse effects of NSAIDs on the course of pneumonia may be specific to bacterial infections. A recent study on the risk of adverse outcomes in users of NSAIDs hospitalized for influenza, found no association between use of NSAIDs and intensive care unit admission or death [9], similar to the findings in this study.”*

4. There is no substantial limitations paragraph with the usual dose, duration etc discussion.

**Author’s response:**
We have expanded and revised the paragraph regarding misclassification of NSAID exposure in the discussion. Please see out respons to the editorial comments for detailed changes.

J Stebbing

-----------------------------------------------------------

Reviewer #2: I confine my remarks to statistical aspects of this paper. These were very well done and I recommend publication.

Peter Flom

**Author’s response:**
Thank you for the positive comments. We are pleased to hear that you found our study fit for publication.

-----------------------------------------------------------

Reviewer #3: This is an interesting and relevant observational study to estimate if there is any effect on patients who obtained a prescription for NSAIDs 30 days prior to a positive SARS-CoV-2 PCR test on mortality within 30 days (primary outcome) or hospitalisation, ICU admission, ventilation or acute renal replacement therapy within 14 days. This has been the subject of much debate during the current COVID-19 outbreak so represents a valuable contribution to the field and will likely impact on clinical management or public health policy.

The set of baseline covariates were defined (age, gender, comorbidities, use of other selected medications plus phase of the outbreak) and the methodology is appropriate. Propensity scores were used to ensure that the 'treated' subjects are similar in their baseline covariates to 'untreated' subjects (matched 1: 4 in this case) in order to provide an unbiased estimate of the relative risk of NSAIDs on the measured outcomes. Additional stratification looked at age (<65y; >65y), gender, cardiovascular disease and non-healthcare worker.

The analyses do not indicate that NSAIDs presented an increased risk of mortality or morbidity in the COVID-19 population in Denmark.

**Author’s response:**
Thank you for the positive comments. We are pleased to hear that you found our study both interesting and relevant.

Best regards,

Anton Pottegård, DMSc
Corresponding author
